# Eras of Digital Entrepreneurship: Connecting the Past, Present, and Future

**DOI:** 10.1007/s12599-021-00728-6

**Published:** 2021-12-06

**Authors:** Tobias Kollmann, Lucas Kleine-Stegemann, Katharina de Cruppe, Christina Then-Bergh

**Affiliations:** 1 Chair of Digital Business and Digital Entrepreneurship, University of Duisburg-Essen, Universitaetsstr. 9, 45141 Essen, Germany; 2 Institute for Entrepreneurship, University of Münster, Geiststraße 24-26, 48151 Münster, Germany

**Keywords:** Digital entrepreneurship terminology, Scoping literature review, Historical eras, Cross-mentions

## Abstract

While recent research continues to emphasize the importance of digital entrepreneurship, the historical terminology of this field is often overlooked. Digital entrepreneurship tends to be considered a new phenomenon despite emerging in the early 1990s. Building on a scoping literature review, this study analyzes 1354 publications that use nine different terms interchangeably to describe the phenomenon of digital entrepreneurship. Based on the number of publications per year, three eras in the historical development of digital entrepreneurship research are outlined. Digital technologies are identified as external enablers, and certain practical events are considered to be influencing factors. The results show that recent research has not adequately recognized the contributions of previous publications and that the understanding of digital entrepreneurship is quite similar with regard to the terms used and over time. This study shows how emerging digital technologies, such as artificial intelligence, blockchain technology, and big data analytics, might shape the future of digital entrepreneurship research. The study occupies the intersection between entrepreneurship and information systems literature and its main contribution is to provide new insights into the eras of digital entrepreneurship from the past to the present and into the future.

## Introduction

Since the mid-1990s, the steady development of digital technologies has enabled not only the creation but also the scaling of so-called digital ventures, whose business models are based on generating value through electronic information via data networks (Kollmann 2006). Against this background, the field of digital entrepreneurship^1^ describes the dovetailing of digital technologies and entrepreneurship (Nambisan 2017). Digital technologies comprise “products or services that are either embodied in information and communication technologies or enabled by them” (Lyytinen et al. 2016, p. 49). Today, the field of digital entrepreneurship has become increasingly important and is a topical issue in both practice and research (Nambisan 2017; Kraus et al. 2019; Ghezzi and Cavallo 2020). In practice, software-based businesses (Alt et al. 2020) using digital technologies as the core of their business models, such as Google, Amazon, Facebook, Apple, and Microsoft (GAFAM), have become the most valuable firms in the world in terms of brand value and market capitalization (Murphy et al. 2020; Swant 2020), underlining the importance of data and information as critical success factors (Weiber and Kollmann 1998; Kraus et al. 2019). Inspired by practical developments, such as the increasing value of the GAFAM firms, the relevance of the field of digital entrepreneurship also continues to grow in research, as shown by the number and quality of publications in highly ranked entrepreneurship and information systems journals (e.g., Ojala 2016; Smith et al. 2017; Srinivasan and Venkatraman 2018; Nambisan et al. 2019; Block et al. 2020).

However, while there has been a pronounced interest in literature on the topic of digital entrepreneurship today, this area has its origin in the emergence of internet technology as the first relevant enabler of digital venture creation (Kollmann 1998; Kollmann et al. 2009). Early developments in internet technology prompted conceptual and empirical research into digital ventures (e.g., Poon and Swatman 1997; Kollmann 1998). In this context, previous literature features several terms, including “internet entrepreneurship,” “e-entrepreneurship,” and “techno-entrepreneurship,” which have often been used as synonyms for “digital entrepreneurship,” leading to confusion over the years (Zaheer et al. 2019). Nevertheless, most studies attempting to characterize this research field have overlooked the longitudinal evolution of terminology and focused on digital entrepreneurship in isolation, referring to it as if it were an emergent and barely researched field (e.g., Grégoire and Shepherd 2012; Kraus et al. 2019). This article problematizes the in-house assumption^2^ that digital entrepreneurship is a new phenomenon (Sandberg and Alvesson 2011; Alvesson and Sandberg 2011, 2014) and explores its evolution. Accordingly, this study seeks to answer the following questions: (1) What is the terminological history of digital entrepreneurship and what role do digital technologies play in it? (2) How are the different terms in the field of research on digital entrepreneurship connected? (3) How have the definitions in the field of digital entrepreneurship changed over time? (4) What are the possible avenues for future research in digital entrepreneurship based on digital technologies?

Building on a scoping literature review (Templier and Paré 2015), we challenge the implication in the existing literature that digital entrepreneurship is a new phenomenon (e.g., Grégoire and Shepherd 2012; Kraus et al. 2019). In the process, we demonstrate how the different terms around digital entrepreneurship have developed over time, enabled by innovative digital technologies and influenced by certain practical events since the early 1990s. We illustrate the role of digital technologies in entrepreneurship (Shen et al. 2018) and show how the different terms are connected by analyzing cross-mentions among publications. Furthermore, we demonstrate how the phenomenon can be defined with reference to the terms used. We then illustrate whether and why these definitions have changed over time. Finally, we identify critical digital technologies that could be sources of new terms and thus enable future eras of digital entrepreneurship research.

We contribute to the literature on digital entrepreneurship in multiple ways (e.g., Davidson and Vaast 2010; Nambisan 2017; Sussan and Acs 2017; Block et al. 2020). First, we provide new insights into the history of today’s digital entrepreneurship terminology based on the specific number of publications per term and year. We show that the publications that did most to drive the development of research on digital entrepreneurship appeared from the early 1990s, following the development and spread of relevant technologies, such as internet technology. Second, we outline the intensity of the connections among the different terms used most frequently within the field. We show that most publications rarely mention other terms, and only two pairs of terms mention each other slightly more often. In addition to exploring the use of terms, we delve deeper into their understanding, showing that they can be interpreted synonymously. Third, we show that some preliminary definitions have evolved over time, leading to the assumption that they reflected the same understanding over time. Therefore, we try to establish box-changing research, motivating other scholars to “reach [] outwards for new ideas, theories, and methods” (Alvesson and Sandberg 2014, p. 980) and integrate further terms into their research. Fourth, we provide new insights into the future of digital entrepreneurship research. Specifically, we demonstrate that, among others, artificial intelligence, blockchain technology, and big data analytics might be future digital technologies capable of facilitating numerous new research opportunities and shaping the terms used in the research field of digital entrepreneurship.

## Method

This study uses a scoping literature review to identify the full extent, range, and nature of the available literature on the topic (Paré et al. 2015; Schryen et al. 2020). The process thus illuminates the historical development of the field of digital entrepreneurship and its terminology. Drawing on the methodological strategy of Arksey and O’Malley (2005) and Templier and Paré (2015), this scoping literature review can be divided into three overarching phases in terms of (1) planning, (2) conducting, and (3) reporting. The method is intended to ensure transparency and reproducibility (Fisch and Block 2018; Keding 2021), which are the most important elements of a trustworthy literature review (Cram et al. 2020). The three steps are described below.

First, as different terms have been used synonymously to describe digital entrepreneurship, resulting in confusion (Matlay 2004; Zaheer et al. 2019), we ensured our analysis included multiple search terms so as to cover the entire field. Several pilot searches and exploratory readings revealed the most important terms in the field of digital entrepreneurship to be “e-entrepreneurship,” “digital entrepreneurship,” “virtual entrepreneurship,” “online entrepreneurship,” “cyber entrepreneurship,” “internet entrepreneurship,” “IT entrepreneurship,” “e-commerce entrepreneurship,” and “techno-entrepreneurship.”

Second, we obtained our data by focusing on the most important databases in the entrepreneurship literature, such as Business Source Premier via EBSCO host and Scopus (Kraus et al. 2020). To ensure we identified every publication that used any of the aforementioned terms in the field of digital entrepreneurship, we considered different spellings and abbreviations. Table 1 illustrates the search terms applied to the titles, abstracts, keywords, and/or subjects of the publications. Using asterisks, we included words that contained not only the term “entrepreneur” but also “entrepreneurial” or “entrepreneurship,” as these words also covered the topic. This search led to a total of 1723 publications. Unlike more traditional systematic literature reviews (e.g., Kraus et al. 2019; Zaheer et al. 2019), our scoping literature review focuses on the breadth of the literature rather than the depth of coverage (Paré et al. 2015). Therefore, our dataset includes all types of publications (e.g., articles, conference papers, book chapters, reviews, and interviews) that used any of the aforementioned terms regardless of the publication’s focus and any quality assessment, such as journal ranking (Anderson et al. 2008). We filtered those publications according to criteria that should ensure the trustworthiness of the dataset and its relevance to the research questions (Templier and Paré 2015; Cram et al. 2020). In particular, we analyzed how prominent a term is in research during a particular period to determine trends in the historical development of digital entrepreneurship. Subsequently, we excluded all publications that were not written in English or published before 1970 because the underlying internet technology that represents one of the cornerstones of digital entrepreneurship did not exist prior to that date (Schatz and Hardin 1994). That process led to 1684 remaining publications. Then, we excluded all existing duplicates for the different terms, leaving a total of 1531 publications. Finally, two authors scanned all titles, abstracts, keywords, and subjects independently to establish the correct use of the terms mentioned above (Paré et al. 2015). They reviewed whether the search terms were mentioned at least once in every publication and referred to the overarching topic of digital entrepreneurship. That review encompassed, for example, ensuring that the term “it entrepreneur*” referred to “information technology” in combination with “entrepreneurship,” rather than a random combination of the words “it” and “entrepreneurship” in general. The final sample comprises 1354 publications produced between 1990^3^ and 2020. Table 1 illustrates the steps undertaken and the precise number of publications connected to each keyword after the consecutive analysis steps.

**Table 1:** Number of publications per keyword

Terms	Database	Number of publications after each filtering step			
		Term in the titles, abstracts, keywords, and/or subjects	Language: english; timeframe: 1970–2020	Exclusion of duplicates	Scanning of titles, abstracts, keywords, and/or subjects
“e-entrepreneur*” OR “electronic entrepreneur*”	EBSCO and Scopus	185	181	167	86
“digital entrepreneur*”	EBSCO and Scopus	383	363	311	306
“virtual entrepreneur*”	EBSCO and Scopus	42	39	34	33
“online entrepreneur*”	EBSCO and Scopus	129	128	110	101
“cyber entrepreneur*” OR “cyberentrepreneur*” OR “cyberpreneur*”	EBSCO and Scopus	35	35	32	25
“internet entrepreneur*” OR “net entrepreneur*”	EBSCO and Scopus	489	486	465	454
“it entrepreneur*”	EBSCO and Scopus	147	145	133	93
“e-commerce entrepreneur*”	EBSCO and Scopus	68	68	59	49
“techno-entrepreneur*” OR “technopreneur*”	EBSCO and Scopus	245	239	220	207
final sample		1723	1684	1531	1354

**Fig. 1:**
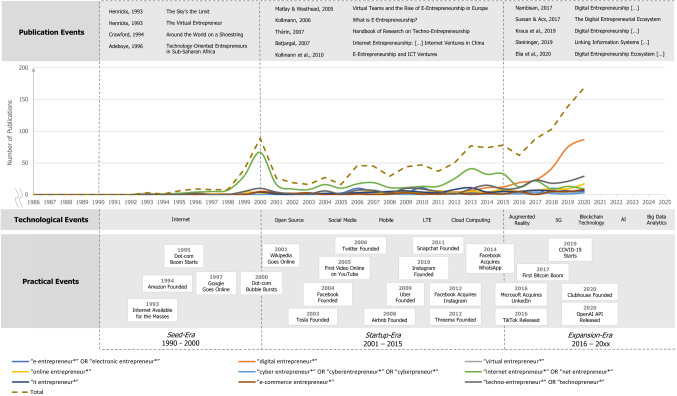
The history of digital entrepreneurship

**Fig. 2:**
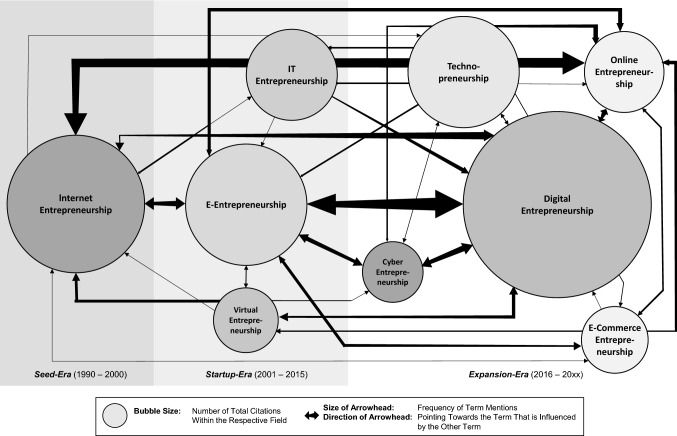
Analysis of cross-mentions

Third, we focused on analyzing and synthesizing the data (Templier and Paré 2015) to present new insights into the history of digital entrepreneurship in a meaningful way (Jesson et al. 2011). We examined the number of publications for each term per year from 1990 to 2020 to understand in which period a specific term was particularly important. Likewise, we identified digital technologies that took on the specific role of enablers for the field of digital entrepreneurship, as well as important practical events that influenced the number of publications. We then matched the number of publications per term with such digital technologies and practical events to show the historical development of digital entrepreneurship along a timeline (see Fig. 1). We also created a net with bubbles positioned chronologically to represent the relevant terms and to illustrate when most publications containing them appeared by year. The size of the bubbles reflects the total number of citations of the respective term field to convey the relevance of those terms to research (Massaro et al. 2016). That number of citations was based on Google Scholar, which offered the only means of identifying up-to-date citations for all articles (Stewart and Cotton 2013). Next, we counted how often publications using one term (e.g., “digital entrepreneurship”) mentioned other terms (e.g., “e-entrepreneurship”) within their titles, abstracts, keywords, subjects, and/or references. We show how the different terms in the field of digital entrepreneurship are connected using arrows between the bubbles, with the size of the arrowheads reflecting on the number of cross-mentions (see Fig. 2).

Furthermore, we attempted to generate further insights into the understanding of the phenomenon over time. Accordingly, we selected the top ten publications per term with the most Google Scholar citations, as this allowed us to generate actuality and comprehensiveness (Stewart and Cotton 2013) and assume that these articles were the most relevant in the respective periods (Massaro et al. 2016). We chose ten publications for each term because we wanted to equally cover every term and include every possible understanding of the phenomenon. The number ten was chosen to encompass publications cited more than 100 times, leading to a sub-set that covers more than half of all citations of the entire dataset (67 percent). We then scanned each of those publications for definitions of the phenomenon, checked whether the terms could be interpreted synonymously with regard to their definition of digital entrepreneurship, and then sorted them chronologically. As many articles did not provide any definitions, our set of articles was reduced by half. Subsequently, we arranged seven of the top definitions as examples to explain the historical development of the understanding of digital entrepreneurship. We chose the seven definitions because they had the most citations on Google Scholar and covered 20 percent of the total citations of our sample while being representative of the definitions during their time. Building on this, we identified links to the number of publications over time and also cross-mentions to provide a distinctive but comprehensive assessment of the understanding of the phenomenon.

Finally, we examined how the research field of digital entrepreneurship might evolve in the future based on the current literature (Schryen 2013; Recker et al. 2019). To do so, we analyzed relevant calls for papers and special issues (Block et al. 2020; Berger et al. 2021), as well as articles with suggestions for future research in the field of digital entrepreneurship (Recker and von Briel 2019; von Briel et al. 2021). We identified, among others, three major developments – artificial intelligence, blockchain technology, and big data analytics – that might lead to new research opportunities and could influence the terms used in future digital entrepreneurship research. For this reason, we searched top-tier journals in the fields of information systems, entrepreneurship, and general/strategic management^4^ for the following word combinations (Steininger 2019): “artificial intelligence” AND “entrepreneur*,” “blockchain” AND “entrepreneur*,” and “big data analytics” AND “entrepreneur*.” We considered articles published only since 2016 to accord with the starting point of the last identified era in this article – the Expansion-Era – and thus the beginning of future digital entrepreneurship research. We checked all articles for content fit and identified 37 articles, providing important insights into possible future eras of digital entrepreneurship.

## Eras of Digital Entrepreneurship: Historical Development

Nambisan (2017) states that digital entrepreneurship describes “the intersection between digital technologies and entrepreneurship” (p. 1029) and addresses the creation and scaling of digital ventures, whose business model is based on generating value through electronic information via data networks (Kollmann 2006). Accordingly, it is a field instigated by the advent of internet technology and has a long history. This study identifies three eras in the historical development of digital entrepreneurship: the Seed-Era (1990–2000), the Startup-Era (2001–2015), and the Expansion-Era (2016–20xx). Every identified era is enabled by innovations in digital technologies and influenced by particular practical events that can explain certain peaks in the number of publications within an era. Figure 1 summarizes the number of publications per term matched with the respective digital technologies and practical events.

### The Seed-Era (1990−2000)

The Seed-Era marks the beginning of historical development in the field of digital entrepreneurship and is primarily characterized by the establishment of internet technology. After about 20 years of development, this technology was finally accessible to the general populace in 1993 (Schatz and Hardin 1994). The fundamental advantages of internet technology, especially in terms of efficiency and effectiveness (Weiber and Kollmann 1998), enabled a wide range of entrepreneurial opportunities through “doing business electronically” (European Commission 1997, p. 2). The first developments in the field of the “internet economy” (Feindt et al. 2002, p. 51) were accompanied by emerging research on these topics (Kollmann 1998). The first terms to describe the impact of internet technology on the field of entrepreneurship were “virtual entrepreneurship,” used in the publications of Henricks in 1993 (1993b, a), and “digital entrepreneurship,” used by Rosenbaum and Cronin (1993). Other terms, such as “internet entrepreneurship” (e.g., Crawford 1994) and “technopreneurship” (e.g., Adeboye 1996), were also used. The appearance of those terms shows that certain pioneers planted the seed – giving this era its name – for this field of research. However, during this period, no term could acquire far-reaching acceptance.

By the late 1990s, the initial opportunities provided by the Internet had been explored, and new business opportunities had emerged (Kollmann 1998). Both practitioners and theorists were confident then, referring to the start of a “promising revolution” (Kollmann 1998, p. 44). Therefore, the *new economy* was defined by the emergence of ever more companies creating electronic value through information via data networks (Weiber and Kollmann 1998; Shapiro et al. 1999; Amit and Zott 2000; Kollmann et al. 2016) including Amazon and Google by the late 1990s. Rather than relying on business models built on traditional value chains (Porter 2001), these companies understood at an early stage the potential of business models built on electronic value (Amit and Zott 2001), leading to the so-called dot-com boom (Senn 2000; Ofek and Richardson 2003).

However, in 2000, the dot-com bubble burst (McFedries 2002), causing investors to lose the money they had staked on the share prices of Internet companies continuing to rise (Zook 2008). In research, the overall peak of publications was reached during the same year with a total of 89 publications, 67 of which used the term “internet entrepreneurship” (see Fig. 1). This peak also marked the end of the Seed-Era as the number of publications reached a turning point. The most frequently used term during the Seed-Era was “internet entrepreneurship” (in 115 out of 163 publications), corresponding to the availability of internet technology that made research in this field possible in the first place. This finding further reinforces how internet technology shaped this era.

### The Startup-Era (2001–2015)

The Startup-Era is one of transition that saw the emergence of many new ways of using internet technology. Examples include new digital technologies, such as open source, social media platforms, mobile, LTE, and cloud computing. After a short recovery period following the bursting of the dot-com bubble, users quickly accepted the new market developments, while new platforms offered them not only more ways to interact with one another via electronic data networks (Cormode and Krishnamurthy 2008; Kollmann et al. 2016) but also the option to take a more active part in the Internet and share almost all forms of data (Richter et al. 2017).

In research, the beginning of the Startup-Era was initially characterized by a significant reduction in publications, most likely owing to the collapse of the dot-com bubble. During the entire era, the number of publications increased only very slowly, and the publication peak of 89 publications in 2000 was never achieved. The analysis of terms used during this second era (15 years in total) shows that the term “internet entrepreneurship” remained the most used (mentioned in 274 out of 631 publications in total); however, other terms, such as “technopreneurship” (88 publications) and “e-entrepreneurship” (59 publications), were gaining traction. Such usage of multiple terms during the era, in the sense of an identification phase, reflects the status quo in practice.

The various terms used in publications in the Startup-Era (e.g., “internet entrepreneurship,” “e-entrepreneurship,” or “technopreneurship”) mainly focused on the digitalization of business processes (e.g., value chains), business models (e.g., Veit et al. 2014), and business environments (Kollmann 2006; Thérin 2007). In this context, research increasingly considered the interconnectivity and networks between actors (e.g., Matlay and Westhead 2005; Gruber and Henkel 2006; Steinberg 2006; Batjargal 2007; Häsel et al. 2010). This also reflected a development in practice – the increase in the involvement of users with the Internet (Provost and Fawcett 2013).

Compared to the Seed-Era, the Startup-Era was characterized by a partial rethinking. In research, discourse on the role of new opportunities, such as open-source software based on internet technology, especially in the field of entrepreneurship, slowly increased. An example was Gruber and Henkel (2006) reflecting on how the domain of open-source software would affect new venture creation processes. Other studies addressed similar aspects (e.g., Zutshi et al. 2006; von Kortzfleisch et al. 2010). However, research on the impact of digital technologies and the new possibilities they engendered remained scarce. Even highly ranked academic journals did not publish articles dealing with this topic, which is why studies increasingly appeared in practice-oriented handbooks (e.g., Thérin 2007; Kollmann et al. 2010).

While the Seed-Era was marked by the domination of the term “internet entrepreneurship,” there was no such clearly dominant term during the Startup-Era. This result corresponds with the finding that internet technology and its various emerging opportunities remained the focus, evidenced by no other single outstanding digital technology emerging to enable a new research direction in this era. In addition, after the dot-com crash at the beginning of this era, no further practical event catalyzed any extraordinary increase or decrease in the number of publications during the Startup-Era.

### The Expansion-Era (2016–20xx)

The last era from 2016 to 20xx is characterized by a turbulent turnaround and the arrival of many new digital technologies that are penetrating the global market (Rippa and Secundo 2019; Kollmann 2020a, b). These technologies introduce digitalization into every aspect of people’s lives. In this context, the processing of large amounts of data (i.e., big data), now underpins many new digital technologies (Dhar et al. 2014; Kollmann 2019), as is particularly evident in the power of the five GAFAM firms, which dominate the collection, processing, and transfer of large amounts of electronic information (Marr 2016).

Similar disrupting developments have also been reflected in research. Although the number of publications initially declined from 78 in 2015 to 62 in 2016, 2017 saw an increase to 88. Interestingly, and yet differing from the previous eras, the frequency of publications focusing on the term “internet entrepreneurship” decreased steadily, whereas publications using the term “digital entrepreneurship” increased (see Fig. 1). This can be identified as a result of the emergence of new digital technologies during this era.

At the same time, research is again subject to reappraisal. The growing popularity of emerging digital technologies has caused scholars to focus on the link between digital technologies and entrepreneurship under the guise of the term “digital entrepreneurship,” and to recognize that “digital technologies are not merely a context in studying entrepreneurship” (Zaheer et al. 2019, p. 2) but “serve as an active ingredient” (Nambisan et al. 2019, p. 2). An increasing number of publications place digital technologies center stage by integrating them into a framework encapsulating digital entrepreneurship (Recker and von Briel 2019) and even creating digital entrepreneurship ecosystems (Sussan and Acs 2017; Elia et al. 2020).

As it turns out, the field of digital entrepreneurship is increasingly being seen as a holistic research domain in its own right. In this holistic system, in which digital technologies are considered ubiquitous (Steininger 2019), scholars acknowledge the growing popularity of digital technologies and attempt to include every aspect of them and explore entrepreneurship in a digital context (Nambisan 2017). There is as yet no sign of that approach abating. At the same time, since 2020 the emphasis on digital technologies has been fueled by the COVID-19 pandemic. While the resulting economic crash reached levels unseen since the great depression of the 1930s, the use of digital technologies and internet traffic increased by about 60 percent (Soto-Acosta 2020). The global pandemic has also affected research and led to conferences and workshops adopting virtual formats (e.g., van der Aalst et al. 2020). However, the boundaries of entrepreneurship are increasingly blurred, as reflected in a trend for digital technologies facilitating what has been termed “everyday everyone entrepreneurship” (van Gelderen et al. 2021, p. 1260), allowing each individual to exploit opportunities and be an entrepreneur. That development has, in turn, led to an evolution of the entrepreneurship phenomenon as a whole.

## In-Depth Analysis of Digital Entrepreneurship

This study now moves on from outlining the historical development of individual terms throughout the three eras to analyze the phenomenon of digital entrepreneurship in greater depth. The aim is to provide an overview of how the different terms are connected and how the understanding of digital entrepreneurship has developed over time. From this, we will present some ideas on the future of digital entrepreneurship research based on relevant digital technologies.

### Cross-Mentions of the Different Terms in the Field of Digital Entrepreneurship

Given that all the described terms are used interchangeably (e.g., Zaheer et al. 2019), we assume that publications using these different terms frequently refer to one another. We thus examined so-called cross-mentions, that is, how often one term (e.g., “e-entrepreneurship”) appeared in publications that used another term (e.g., “digital entrepreneurship”). Our analysis reveals that publications utilizing one term mention other terms only 168 times in the entire dataset (*n* = 1354). Nevertheless, all terms are mentioned at least once by publications that use another term (see Table 2).

**Table 2:**
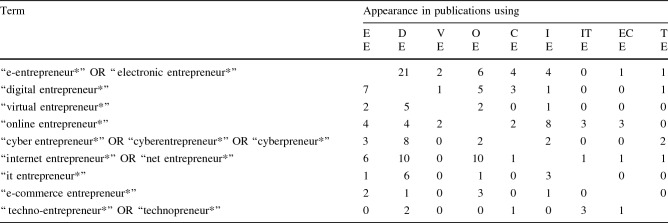
Cross-mentions among publications

The frequency with which the various terms appear in publications utilizing another term varies (from 0 to 21 times), resulting in distinct levels of connectivity. Figure 2 illustrates the strength of connections among the different terms (bubbles) by the size of the arrowhead, which is based on the frequency of cross-mentions.

Our results show that most mentions occur bilaterally, whereas only eight mentions occur unilaterally. The majority of all mentions occur only in the references (116 times) and far less often in titles, abstracts, and keywords (52 times). Based on the strength of connections, two particularly stand out: the link between “online entrepreneurship” and “internet entrepreneurship” and that between “e-entrepreneurship” and “digital entrepreneurship.” While most cross-mentions between other fields appear only between one and five times, these terms are mentioned between seven and 21 times in the field of the other term. Consequently, we investigate these in terms of quantity, content, and time.

It is evident that the connection among publications using the terms “digital entrepreneurship” and “e-entrepreneurship” is stronger but also more asymmetrical; that is, publications dealing with “digital entrepreneurship” mention the term “e-entrepreneurship” three times more often than vice versa (see Table 2). Second, our results indicate that the forms of the mentions are different. “Online entrepreneurship” and “internet entrepreneurship” are often used in titles, abstracts, and keywords (13 times) and rarely appear in the references (five times). In contrast, the terms “e-entrepreneurship” and “digital entrepreneurship” are mostly used only in the references (24 times) and rarely appear in titles, abstracts, and keywords (four times). Moreover, while the terms “internet entrepreneurship” and “online entrepreneurship” are often used synonymously (e.g., Dobbs and Buelow 2000; Peng and Chen 2012; Dai et al. 2018), publications dealing with “digital entrepreneurship” use the term “e-entrepreneurship” to establish demarcation, that is, to actively present the term “digital entrepreneurship” as a new area of research. The only publication that actively uses this term in the abstract calls “digital entrepreneurship” a further development of “e-entrepreneurship” (Gagan et al. 2018), contradicting the existing otherwise interchangeable usage. Third, the number of mentions among the terms regarding the era in which they are mentioned differs. In the term field of “online entrepreneurship,” the most mentions by publications dealing with “internet entrepreneurship” appear in the Expansion-Era (five times) and the Startup-Era (four times) and vice versa (four times in the Startup-Era and three times in the Expansion-Era). The comparable number of mentions in these eras could be explained by the largely synonymous use of these terms.

In contrast, publications on “digital entrepreneurship” mention the term “e-entrepreneurship” most often in the Expansion-Era (16 out of 21 times) and vice versa (five out of seven times). These results show that “e-entrepreneurship,” which belongs to the Startup-Era, is still frequently mentioned in the Expansion-Era. This finding indicates that “e-entrepreneurship” or other terms used in previous eras enrich other terms used today and are therefore highly relevant when investigating the topic of digital entrepreneurship.

### Defining Digital Entrepreneurship Over Time

The eras of digital entrepreneurship and the cross-mentions confirm that research in this field has been conducted since 1990. However, other terms dominated before the term “digital entrepreneurship” gained traction in current studies, which supports our problematization (Sandberg and Alvesson 2011; Alvesson and Sandberg 2011, 2014) that digital entrepreneurship is not a new phenomenon. To advance the “dialectical interrogation” (Alvesson and Sandberg 2011, p. 252), we need to determine how digital entrepreneurship has been understood over time. The definition changing significantly, for example, would justify it being designated a new phenomenon. Accordingly, we examined the most-cited articles for all nine terms to identify definitions reflecting the understanding of digital entrepreneurship.

To enhance the understanding of how digital entrepreneurship evolved, we initially considered all terms together and analyzed definitions irrespective of the terms used. We found that the majority of publications assumed the term was well known and thus did not define it (e.g., Gould and Zhao 2006; Batjargal 2007). When definitions appeared, they might be implicit, as in the work of Bolton and Thompson (2004) that defined both “entrepreneurship” and “internet business” but did not combine the two into a single definition, such as one for “internet entrepreneurship.” The remaining articles that defined the field of digital entrepreneurship (Kollmann 2006; Hull et al. 2007; Davidson and Vaast 2010; Nambisan 2017; Sussan and Acs 2017) reveal that the phenomenon is often understood similarly, even if a certain development over time can be identified. The point is exemplified by the seven example definitions listed in Table 3. First, we found some general definitions, which were mostly published in the Startup-Era. These were rather general and universal. They claimed that some or even all transactions had to be shifted to the digital sphere (e.g., Matlay and Westhead 2005; Gruber and Henkel 2006; Hull et al. 2007) and assumed a “purely electronic creation of value” (Kollmann 2006, p. 333). In this context, all authors explicitly mention the relevance of the Internet as an enabling technology. Second, we identified some expanded definitions, most of which were published in the Expansion-Era. These were not only cited more often (e.g., Nambisan 2017; Sussan and Acs 2017), but they also provided more fine-grained definitions. Davidson and Vaast (2010), for example, define digital entrepreneurship as the “pursuit of opportunities based on the use of digital media and other information and communication technologies” (p. 2), which thus matches the main characteristics of entrepreneurship (Bolton and Thompson 2004) with digital technologies. Other scholars have followed this dichotomy, such as Sussan and Acs (2017) and Nambisan (2017), who call digital entrepreneurship “the intersection of digital technologies and entrepreneurship” (p. 1029).

**Table 3:** Definitions of digital entrepreneurship over time

	Author(s)	Definitions
General definitions	Matlay and Westhead (2005)	Recent research has established that e-entrepreneurs differ from their traditional counterparts in that all of their economic transactions take place online, via the Internet (Chulikavit and Rose 2003; Matlay 2003a, b). (p. 282)
	Kollmann (2006)	E-entrepreneurship refers to establishing a new company with an innovative business idea within the net economy, which, using an electronic platform in data networks, offers its products and/or services based upon a purely electronic creation of value. Essential is the fact that this value offer was only made possible through the development of information technology. (p. 333)
	Gruber and Henkel (2006)	The term “e-entrepreneurship” has been coined to address the discovery and exploitation of business opportunities in the internet economy. (p. 1)
	Hull et al. (2007)	Digital entrepreneurship is a subcategory of entrepreneurship in which some or all of what would be physical in a traditional organization has been digitized […]. This entrepreneurial activity relies on information technology to create, market, distribute, transform or provide the product. (p. 293)
Expanded definitions	Davidson and Vaast (2010)	We refer to digital entrepreneurship as the pursuit of opportunities based on the use of digital media and other information and communication technologies. Digital entrepreneurs rely upon the characteristics of digital media and IT to pursue opportunities […]. The term digital entrepreneurship encompasses the diverse opportunities generated by the Internet, World Wide Web, mobile technologies, and new media. (p. 2)
	Sussan and Acs (2017)	[Digital entrepreneurship] is the combination of digital infrastructure and entrepreneurial agents within the context of both ecosystems. […] (p. 66)
	Nambisan (2017)	In recent years, the infusion of new digital technologies […] into various aspects of innovation and entrepreneurship has transformed the nature of uncertainty inherent in entrepreneurial processes and outcomes as well as the ways of dealing with such uncertainty. In turn, this has opened up a host of important research questions at the intersection of digital technologies and entrepreneurship – on digital entrepreneurship. (p. 1029)

We next analyzed all definitions within the context of their term field to show the possible differences between terms. Our analysis supports the findings by Zaheer et al. (2019) that these terms can be understood synonymously.^5^ Nevertheless, the varying degrees of mentions of other terms are also noticeable in the definitions. With regard to the term field of “e-entrepreneurship,” for example, Matlay and Westhead (2005) refer to earlier, and not directly associated, sources and even admit that the related “term […] ‘e-Economy’ [was] often used interchangeably with ‘Digital Economy’” (p. 280). Gruber and Henkel (2006) use references dealing with digital phenomena in the 1990s (i.e., Weiber and Kollmann 1998) as part of their expanded definition, which is less common in the term field of “digital entrepreneurship.” Here, it can be seen that publications mostly try to establish their own definitions without mentioning prior work (e.g., Davidson and Vaast 2010), even if their definitions are often similar to earlier ones from publications on, for example, “e-entrepreneurship.” Some recent research publications do refer to earlier works but either build upon definitions from the same term field (e.g., Kraus et al. 2019) or refer to earlier works published using the same term (e.g., Dy et al. 2017).

Overall, it is evident that digital entrepreneurship has been understood in very similar ways, not only within the framework of the various terms but also over time. However, after some general definitions were provided that offered a basis for future work, there was a shift toward redefining the phenomenon rather than referring to older definitions. This process was accompanied by an increasingly differentiated examination of the phenomenon itself. While initially the Internet – the dominating technology of the time – was assumed to be the sole source of digital entrepreneurship, today, the understanding is far more multifaceted, and not only in terms of the technology itself. Some studies now differentiate digital entrepreneurship in relation to digital technologies according to the former’s roles or functions. Steininger et al. (2019) create categories based on digital technology serving as a facilitator, mediator, outcome, or ubiquity. In contrast, Sahut et al. (2019) differentiate between a function as an enabler and the function as both an output and enabler of digital entrepreneurship. Other scholars have established new subcategories that shape the phenomenon, including Nambisan (2017) who proposes a division between digital artifacts, platforms, and infrastructures, which are interrelated but have different implications for digital entrepreneurship. Giones and Brem (2017) identify further subcategories of the phenomenon itself depending on the digital technologies used, stating that “[w]e have reached a consolidation stage in technology entrepreneurship research” (p. 44). Now that the preliminary work to define digital entrepreneurship is complete, it is often more a matter of refining an existing field or unveiling new aspects than of redefining the phenomenon.

### The Future of Digital Entrepreneurship Research

This study reveals that digital entrepreneurship has a longer and more eventful history than is often assumed. The findings indicate that digital technologies are particularly productive sources of new terms and eras in the research field of digital entrepreneurship. An examination of current research (Schryen 2013; Recker et al. 2019) helps identify artificial intelligence, blockchain technology, and big data analytics as decisive digital technologies that enable the future of digital entrepreneurship research (see Method). Table 4 presents potential future research directions in the field of digital entrepreneurship based on illustrative studies within each digital technology in the entrepreneurship context.

**Table 4:** The future of digital entrepreneurship and possible research opportunities

The future of digital entrepreneurship	Possible research directions	Illustrative studies
AI-Entrepreneurship	Artificial intelligence and… Entrepreneurial opportunities Entrepreneurial decision making Future business models (Team) Processes Entrepreneurial rewards Entrepreneurial ecosystems Entrepreneurial financing Entrepreneurial research/education	Garbuio and Lin (2019), Elia et al. (2020), Prüfer and Prüfer (2020), Liebregts et al. (2020), Obschonka and Audretsch (2020), Obschonka et al. (2020), Palmié et al. (2020), Chalmers et al. (2020), Fossen and Sorgner (2021), Hannigan et al. (2021), Korzynski et al. (2021), Robledo et al. (2021)
Blockchain-Enabled/Supported Entrepreneurship	Blockchain technology and… Entrepreneurial financing (e.g., ICOs) Cryptocurrencies (e.g., Bitcoin) Compliance standards and contracts Business models Electronic marketplaces Innovation (e.g., intellectual property) Transaction costs	de Soto (2017), Fisch (2019), Ahluwalia et al. (2020), Allen et al. (2020), Huang et al. (2020), Kollmann et al. (2020a, b), Masiak et al. (2020), Momtaz (2020), Bellavitis et al. (2020), Bogusz et al. (2020), Chang et al. (2020), Chalmers et al. (2020, 2021), Islam et al. (2021), Kher et al. (2021), Meier and Sannajust (2021), Schückes and Gutmann (2021), Toufaily et al. (2021), Zanella et al. (2021), Zheng et al. (2021), Block et al. (2021)
Data-Driven Entrepreneurship	Big data analytics and… Business model innovation Entrepreneurial opportunity evaluation Strategic orientation Innovation analytics	Çanakoğlu et al. (2018), Lévesque and Joglekar (2018), Lin and Kunnathur (2019), Ciampi et al. (2021), Mariani and Nambisan (2021)

First, the advance of artificial intelligence is one of the greatest technological revolutions of our time (Makridakis 2017). Understanding how algorithms perform tasks or resolve complex problems, traditionally solved by human intelligence, might lead to disruptive changes in various disciplines, such as economics (e.g., Brynjolfsson and Mitchell 2017), policy (e.g., Agrawal et al. 2019), management (e.g., Keding 2021), innovation (e.g., Aghion et al. 2017), and psychology (e.g., Glikson and Woolley 2020). Against this backdrop, scholars have recently begun to consider the interplay between artificial intelligence and entrepreneurship on a conceptual and empirical basis (e.g., Obschonka and Audretsch 2020; Chalmers et al. 2020). Researchers anticipate that the automation ability of artificial intelligence and its predictive capabilities will affect opportunity recognition, evaluation, and exploitation (Shane and Venkataraman 2000) at all stages of the entrepreneurial process (Garbuio and Lin 2019; Fossen and Sorgner 2021). Artificial intelligence could also change current or future business models (Chalmers et al. 2020) and affect future entrepreneurial decision-making (Liebregts et al. 2020) and the entrepreneurial ecosystem as a whole (Elia et al. 2020). Consequently, we anticipate that improvements in artificial intelligence could define one of the forthcoming eras in the field of digital entrepreneurship, for example, by using the term “AI-entrepreneurship” (Chalmers et al. 2020).

Second, developments in blockchain technology might reveal new opportunities for future digital entrepreneurship (e.g., Nofer et al. 2017; Nambisan et al. 2019; Rippa and Secundo 2019). For example, artificial intelligence–blockchain hybrid platforms could help new ventures address the challenges that they typically face in their early development stages, such as managing financial accounting, compliance standards, and legal work (Chalmers et al. 2020). Cryptocurrencies, such as Bitcoin or Ethereum, and the associated blockchain technology might provide new payment options for digital products and services (Masiak et al. 2020; Momtaz 2020); e.g., (Kher et al. 2021). Cryptocurrency might also open access to external capital for digital ventures in the form of an initial coin offering (e.g., Fisch 2019; Ahluwalia et al. 2020; Bogusz et al. 2020; Huang et al. 2020). Blockchain technology might spur new digital business models (Bellavitis et al. 2020), for instance, by replacing typical intermediaries in electronic marketplaces (Kollmann et al. 2020a, b). Therefore, blockchain technology might act as an external enabler of future digital entrepreneurship, leading to “blockchain-enabled entrepreneurship” or “blockchain-supported entrepreneurship” (Chalmers et al. 2021).

Third, having access to big data and being able to analyze them could become increasingly important to entrepreneurs and their ventures (Berg et al. 2018; Kleine-Stegemann 2021). The development of big data analytics capabilities, considered as a “company’s abilities to leverage on technology and talent to exploit big data” (Ciampi et al. 2021, p. 2) – could therefore be critical for entrepreneurial actors to compete in highly dynamic and digitalized markets. Individuals and organizations with big data analytics capabilities are the most likely to exploit the potential to reduce entrepreneurial risks and uncertainties (Çanakoğlu et al. 2018), inform entrepreneurial decisions (Lévesque and Joglekar 2018), and improve venture innovation performance (Mariani and Nambisan 2021), for instance. We expect the large amount of data and the burgeoning options to analyze them might lead to “data-driven entrepreneurship,” where data-driven techniques and technologies shape the elements of the entrepreneurial process (Çanakoğlu et al. 2018).

## Discussion

Although research in the field of digital entrepreneurship is of paramount importance in today’s entrepreneurship literature (e.g., Nambisan 2017; von Briel et al. 2018; Block et al. 2020), the terminological history of the field is often overlooked. The present study follows the methodological approach of Alvesson and Sandberg (2011; 2014) to problematize the in-house assumption that digital entrepreneurship is a new phenomenon. We have reviewed the origins of the terms used in the field of digital entrepreneurship and their growth in popularity. Our findings indicate that innovative digital technologies enabled that growth in terms relating to the field of digital entrepreneurship in certain eras. Relevant practical events influenced the number of publications within those identified eras. Moreover, even when terms are used interchangeably, they rarely reference each other, as illustrated by examining the evolution of the definitions, ultimately indicating a very similar understanding of the phenomenon. Our findings support four decisive contributions to theory.

First, our study extends prior findings by Zaheer et al. (2019) by identifying three relevant eras based on our publications analysis per term and year: the Seed-Era (1990–2000), the Startup-Era (2001–2015), and the Expansion-Era (2016–20xx). Distinguishing these three eras allows us to highlight the scientific dependence of entrepreneurship research on key technological developments. While other academic terminologies seem to be driven by regulatory factors, digital entrepreneurship terms are still rooted in practical phenomena (i.e., the development and spread of digital technologies). Accordingly, we add to the scientific debate by demonstrating the relevance of temporal contingencies to the emergence of new research topics and terminologies. This insight might support future studies attempting to bridge the gap between practice and research (Shen et al. 2018) and predict future evolutions in research. Therefore, researchers should always remain abreast of new digital technologies and maintain connections with practice.

Second, our study generates new knowledge on the connections among the different terms of today’s digital entrepreneurship by analyzing how often publications using a certain term (e.g., “e-entrepreneurship”) mention another one (e.g., “digital entrepreneurship”). Surprisingly, our findings reveal that researchers using one term rarely mention another in their published work; if they do so, it is likely to be only in the references. This omission of historical terms could be explained as follows: It could be that at the time of publication researchers were not yet able to generate a deeper understanding of the phenomenon and thus could not recognize that other terms, such as “e-entrepreneurship” and “internet entrepreneurship,” also describe the phenomenon of digital entrepreneurship. Therefore, they unintentionally excluded other terms from their studies. Another reason could be that researchers were aware of the history of the research field, but chose to stick with one term because they considered all other terms to be synonyms (e.g., Elia et al. 2020).

Moreover, some studies might simply not search for historical terms or simply ignore them as an element of a demarcation strategy (e.g., Gagan et al. 2018). Our results indicate that the term “digital entrepreneurship” continues to dominate new publications, which we suggest is a consequence of researchers seeking novelty and uniqueness by establishing a terminological distance from other longer-established terms.

Third, this study reveals new insights into the evolution of the understanding of digital entrepreneurship (e.g., Steininger 2019; Zaheer et al. 2019). We show that despite some changes prompted by the ongoing integration of digital technologies into our lives, the basic understanding has remained consistent. Accordingly, we add to the scientific discourse (e.g., Nambisan 2017; Sussan and Acs 2017; Shen et al. 2018; Steininger 2019; Elia et al. 2020) holding that contingency over time is also highly relevant when considering the content of the phenomenon and that the enabling role of digital technologies is reflected in the basic understanding of it. The current research thus extends previous research, such as that of Giones and Brem (2017) and Sahut et al. (2019), who put a content-based division of digital entrepreneurship center stage.

Fourth, we generate new knowledge about how future research on digital entrepreneurship might look like (e.g., van Gelderen et al. 2021). We identify artificial intelligence (Chalmers et al. 2020), blockchain technology (Chen and Bellavitis 2020; Kollmann et al. 2020a, b), and big data analytics (Çanakoğlu et al. 2018) as potentially groundbreaking digital technologies, thus offering other research topics that subsequent studies might explore. We also apply our findings to the terminological evolution of terms and suggest future terms for digital entrepreneurship based on the underlying digital technologies used.

## Limitations

Despite the study’s contributions, we must acknowledge several limitations regarding the generalizability of our statements. First, we used a large dataset (*n* = 1354). Although this large dataset with few exclusion criteria is typical of scoping literature reviews (Paré et al. 2015), future studies could validate or extend our findings with more traditional systematic literature reviews. For instance, studies could conduct in-depth content analyses only in highly ranked academic journals to generate an even deeper understanding of the history of digital entrepreneurship (Anderson et al. 2008).

Second, we only excluded duplicates within one term field (i.e., if a single publication using “e-entrepreneurship” is listed more than once in the term field of “e-entrepreneurship”) and not between term fields (i.e., if a single publication using “e-entrepreneurship” is listed more than once between the term fields of “e-entrepreneurship” and “digital entrepreneurship”). The approach was dictated by there being no objective decision criteria on which to assign a publication to just one term field when it is mentioned in multiple term fields. The situation means that publications mentioning various terms in their titles, abstracts, keywords, and/or subjects could have appeared in multiple term fields and thus more than once in our overall dataset. Further research could extend our findings by controlling for the possible effects of multiple occurrences of publications between the term fields.

Third, we identify the connections among the different terms of digital entrepreneurship based on the cross-mentions between the publications within titles, abstracts, keywords, subjects, and/or references. Future studies could extend these results by searching for cross-mentions also in the main body of the study or using explorative quantitative methods, such as searching for networks and graph representations of citations between the documents via bibliometric (Zupic and Čater 2015) or network analysis (Bhupatiraju et al. 2012).

Fourth, we have expanded the understanding of digital entrepreneurship based on given definitions. We did not search all publications for definitions but only the top ten per term (which nevertheless covered 67 percent of all citations of the entire dataset). Subsequent studies might consider additional definitions to expand our findings. Furthermore, the understanding of a phenomenon is often reflected in both the definitions employed and other parts of the publication. Future research could include more than just the stated definitions (e.g., the content of the abstract or the body text) to obtain more detailed information on the basic understanding of the publications.

Finally, we determine the future of digital entrepreneurship research based on current calls for papers and future research direction sections within articles in the field of digital entrepreneurship. While our approach facilitates a prediction of the future based on the literature (Schryen 2013; Recker et al. 2019), future research might employ other methods, such as the Delphi method that offers a systematic and multilevel estimation procedure to predict future events (van Gelderen et al. 2021).

## Footnotes

1 In our study, we distinguish between the overall field of research on digital entrepreneurship (i.e., “the field of research on digital entrepreneurship”) and the term “digital entrepreneurship” itself (i.e., “digital entrepreneurship”).

2 Problematization challenges “the assumptions that underlie not only others’ but also one’s own theoretical position and, based on that, to construct novel research questions” (Alvesson and Sandberg 2011, p. 252). In-house assumptions are those that “exist within a particular school of thought in the sense that they are shared and accepted as unproblematic by its advocates” (Alvesson and Sandberg 2011, p. 254).

3 Although internet technology was introduced as early as 1970, it did not become fully accessible to the general population until the early 1990s. This appears to explain why despite searching from 1970 we did not find the first publications until 1990.

4 We included only articles from peer-reviewed journals having the minimum VHB-Jourqual 3 rating of “B.” Jourqual 3 is a magazine ranking published by the German Academic Association for Business Research. It can be accessed online at http://www.vhbonline.org (retrieved on September 9th, 2021).

5 Interestingly, we could also not find any major differences in the understanding between the research fields (i.e., entrepreneurship, technology, and innovation). What was evident, however, was a slightly stronger focus on the technological side of digital entrepreneurship in technology journals (e.g., Giones and Brem 2017).
